# Auditory stimulation during sleep suppresses spike activity in benign epilepsy with centrotemporal spikes

**DOI:** 10.1016/j.xcrm.2021.100432

**Published:** 2021-10-26

**Authors:** Jens G. Klinzing, Lilian Tashiro, Susanne Ruf, Markus Wolff, Jan Born, Hong-Viet V. Ngo

**Affiliations:** 1 Institute of Medical Psychology and Behavioral Neurobiology, University of Tübingen, 72076 Tübingen, Germany; 2 Centre for Integrative Neuroscience, University of Tübingen, 72076 Tübingen, Germany; 3 Princeton Neuroscience Institute, Princeton University, Princeton, NJ 08540, USA; 4 University Children’s Hospital Tübingen, 72076 Tübingen, Germany; 5 Department of Pediatric Neurology, Vivantes Hospital Neukölln, 12351 Berlin, Germany; 6 Department of Psychology, University of Lübeck, 23562 Lübeck, Germany

**Keywords:** BECTS, rolandic epilepsy, spikes, sleep, auditory stimulation, sleep spindles

## Abstract

Benign epilepsy with centrotemporal spikes (BECTS) is a common form of childhood epilepsy linked to diverse cognitive abnormalities. The electroencephalogram of patients shows focal interictal epileptic spikes, particularly during non-rapid eye movement (NonREM) sleep. Spike formation involves thalamocortical networks, which also contribute to the generation of sleep slow oscillations (SOs) and spindles. Motivated by evidence that SO-spindle activity can be controlled through closed-loop auditory stimulation, here, we show in seven patients that auditory stimulation also reduces spike rates in BECTS. Stimulation during NonREM sleep decreases spike rates, with most robust reductions when tones are presented 1.5 to 3.5 s after spikes. Stimulation further reduces the amplitude of spikes closely following tones. Sleep spindles are negatively correlated with spike rates, suggesting that tone-evoked spindle activity mediates the spike suppression. We hypothesize spindle-related refractoriness in thalamocortical circuits as a potential mechanism. Our results open an avenue for the non-pharmacological treatment of BECTS.

## Highlights

Spikes in BECTS epilepsy and sleep spindles may share thalamocortical generation
Auditory stimulation during sleep evokes sleep spindles and suppresses spikes
Stimulation may reduce spiking by inducing thalamocortical refractoriness

## Graphical abstract

**Figure:**
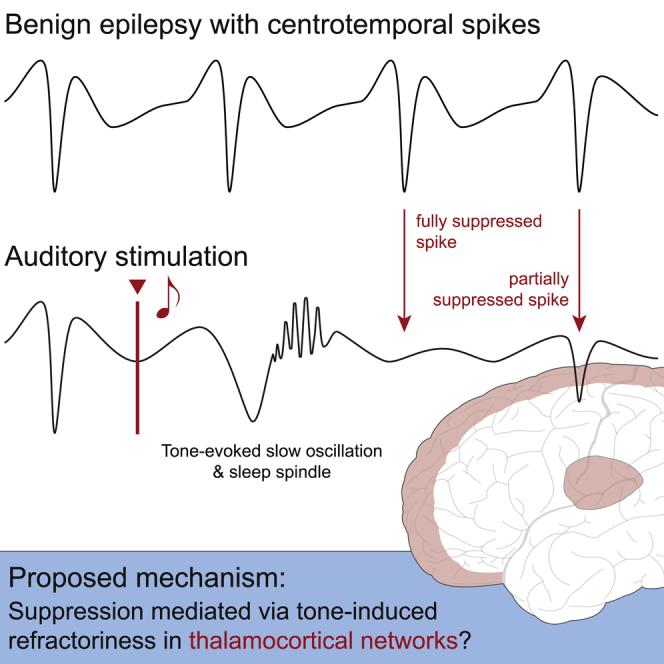

## Teaser

Interictal spikes hallmarking benign epilepsy with centrotemporal spikes (BECTS) have been hypothesized to emerge from thalamocortical networks. These networks likewise generate spindle activity during sleep. Klinzing et al. demonstrate that auditory stimulation during sleep suppresses interictal spikes in BECTS, possibly by inducing refractoriness in shared thalamocortical networks.

## Introduction

Benign epilepsy with centrotemporal spikes (BECTS; also rolandic epilepsy) is the most common form of focal childhood epilepsy.^1^^,^^2^ While its impact on cognitive functions is often mild compared to other sleep epilepsies, such as continuous spikes and waves during sleep (CSWS), BECTS has recently been associated with a number of deficits^3^ such as lowered academic success,^4^ impaired language,^4^^,^^5^ and hampered memory storage and retrieval,^3^ as well as impairments of attention.^6^ Overt seizures occur predominantly around sleep on- or offset^1^ and tend to be rare. However, electroencephalographic recordings show frequent interictal epileptiform discharges (or “spikes”) occurring during sleep and quiet waking.^7^ Spikes are most common during early NonREM (non-rapid eye movement) sleep^8^ and present in the electroencephalogram (EEG) as sharp high-amplitude deflections, sometimes followed by a slower surface negative wave. As indicated by the name BECTS, spikes are typically most prevalent over centrotemporal sites, with neocortical sources clustered around the central gyrus.^9^ The intensity of spiking in BECTS has been suggested to correlate with the severity of cognitive impairments,^10^ and selective cognitive deficits in children with BECTS have been demonstrated to be related to the location of the focal spike.^11^ In other sleep-associated epileptic disorders, e.g., epilepsy with CSWS, the rate of spikes has been linked to disturbed sleep rhythms and functions.^12^^,^^13^

While the primary locus of spike expression is cortical, early evidence indicates that the formation of spikes in BECTS involves thalamocortical connections.^9^^,^^14^, ^15^, ^16^, ^17^ Thalamocortical connections are also involved in the generation of slow oscillatory (SO) activity and sleep spindles.^18^, ^19^, ^20^ These rhythms are hallmarks of NonREM and slow-wave sleep and are essential for plastic processes underlying memory consolidation^21^ as well as the homeostatic regulation of global synaptic strength in cortical networks during sleep.^22^ In BECTS, spikes appear to be most closely linked to spindles,^14^^,^^23^ which, in addition to their role in memory,^19^^,^^20^^,^^24^ also correlate with intelligence.^25^^,^^26^ Pathologies in thalamocortical spindle microcircuits can result in epileptic discharges.^27^^,^^28^ Consequently, epileptic spikes disrupting physiological spindle formation may interfere with the plastic functions of sleep, a mechanism that has been proposed as a cause for cognitive impairments in epilepsy.^29^^,^^30^

The occurrence of sleep spindles in thalamocortical networks is top-downregulated by the neocortical SO, which drives networks of the thalamic nucleus reticularis into spindle-rhythm generation.^19^^,^^31^ As a result, spindles are commonly nested into SO up states, while they are absent during the hyperpolarized down state.^32^^,^^33^ Importantly, SOs and spindles can both be controlled via external auditory stimulation. Presenting single tones during sleep is known to elicit an SO-like wave, the so-called K-complex that typically comprises a spindle during its depolarized up state.^34^^,^^35^ Considering the possible overlaps of thalamocortical circuits involved in the generation of SOs, spindles, and epileptic spikes in BECTS, we hypothesized that auditory stimulation might also modulate spiking activity. We further expected that these interfering effects of stimulation would differ depending on the delay between spike occurrence and tone presentation. To test this, we performed tone stimulations at varying delays from a detected spike. Indeed, we observed the greatest effects with the tones presented at the largest temporal distance to spikes. These results present a potential first step toward a non-pharmacological intervention for BECTS.

## Results

### Auditory stimulation reduces the number of interictal spikes

**Figure 1:**
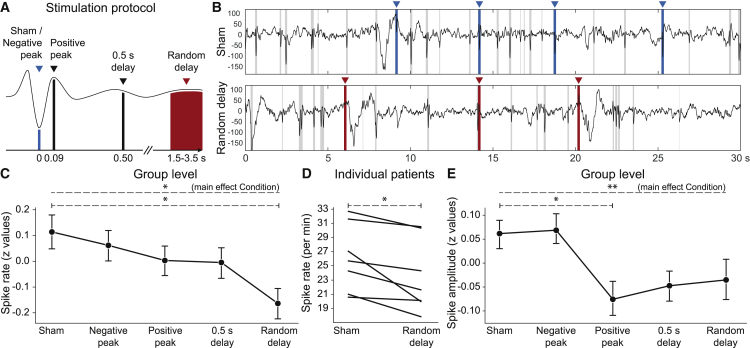
Auditory random-delay stimulation reduced spike rates and amplitudes (A) Auditory stimulation was performed at the time of a spike’s negative peak (“negative peak”), positive peak (by introducing a delay of 90 ms, “positive peak”), 0.5 s after the negative peak (“0.5 s delay”), or with a random delay between 1.5 and 3.5 s after the negative peak (“random delay”). In a “sham” control condition, no stimulus was delivered. The stimulation conditions were conducted in blocks of 30 s. (B) Representative examples for 30 s blocks without stimulation (sham, 26 detected spikes) and random delay stimulation (random delay, 18 detected spikes). Blue and red markers show sham and random delay stimulations, respectively. Grey bars mark detected spikes. (Note, in the random delay condition, the second tone (t = ∼14 s) by chance occurred close to another spike.) (C) The rate of spikes was modulated by the stimulation (ANOVA on normalized and pooled data, within-subject factor “condition,” p = 0.021) and significantly lower in the random delay condition compared to sham (Holm-corrected p = 0.015). (D) Spikes were suppressed in each of the seven patients (paired t test on non-normalized, non-pooled individual data, p = 0.020). (E) The amplitude of spikes right after tones (<1.5 s) was reduced in most stimulation conditions (ANOVA on normalized data, main effect condition, p = 0.002). When compared to sham, this reduction was significant for the positive delay condition (Holm-corrected p = 0.021; 0.5 s delay, p = 0.121; random delay, p = 0.250). Data were taken from the detection electrode. Data in (C) and (E) were normalized across all conditions within each patient, pooled, and represented as mean ± boot-strapped SEM. ∗p < 0.05; ∗∗p < 0.01.

We presented tones (short bursts of pink noise) during NonREM sleep of seven BECTS patients and seven age-matched controls (see Table S1 for further participant details and Table S4 for their sleep parameters in the experimental night). We employed five different closed-loop stimulation protocols (Figure 1A) that varied the time delay to epileptic spikes. These spikes were detected using an amplitude threshold on the bandpass-filtered EEG signal recorded close to the epileptic focus (i.e., the detection electrode, DET). This allowed us to assess the effects of the stimulation on already ongoing as well as subsequent epileptic activity. In three “peri-spike stimulation” protocols, tones were presented either immediately upon detection of a spike (“negative peak” condition), 90 ms later (“positive peak”), or 500 ms later (“0.5 s delay”). In a “random delay” condition, stimulation was performed after a random interval of 1.5 to 3.5 s following the negative peak of the spike. Finally, in a “sham” control condition, spikes were detected, but no stimulation was performed. Protocols were switched randomly after blocks of 30 s. The total stimulation time window (i.e., the sum of all 30 s stimulation blocks) per participant was 100.1 ± 4.3 min. Within that time window, the proportion of spikes that triggered a subsequent stimulation was 35.3% ± 1.9%.

To analyze the effect of stimulation, spike rates were determined for each stimulation block and pooled across all patients (see Figure S1 for the detection procedure). This analysis revealed a significant reduction in spike rates mainly driven by a decrease in the random delay condition (23.316 ± 0.682 events/min) in comparison to the sham condition (26.452 ± 0.764 events/min), corresponding to a decrease of 11.86% (ANOVA main effect Condition, F(4,1328) = 2.615, p = 0.034, η^2^ = 0.008, with Holm-corrected p = 0.015, d = 0.269 for random delay versus sham post hoc test; see Table S2 for spike rates for each patient and condition). To prevent potential baseline differences between subjects to invalidate the analysis, we normalized spike rates and performed statistics again on the resulting *Z*-values (Figure 1C). This analysis resulted in an even stronger effect, showing the robustness of the suppression (ANOVA main effect condition, F(4,1328) = 2.895, p = 0.021, η^2^ = 0.009; Holm-corrected p = 0.015, d = 0.271 for random delay versus sham post hoc test). The significant reduction of spike rates in the random delay condition remained when comparing non-normalized, non-pooled spike rate averages between these conditions (paired-samples t test, p = 0.020, d = 1.189; Figure 1D). Further control analyses showed that, as intended, our protocols led to stimulations that were not systematically phase-locked to ongoing slow waves (Rayleigh test for uniform distribution of stimulation time points across instantaneous slow wave phase, p > 0.343) or ongoing spindle activity (ANOVA on likelihood of spindle occurrence at stimulation time point across conditions, p = 0.252).

While overall, spike amplitude was not altered by the stimulation (ANOVA main effect condition, F(4,16643) = 1.773, p = 0.131), we asked whether spikes that immediately followed a tone (i.e., within 1.5 s) were specifically affected. We found that the amplitude of such spikes was significantly diminished, in comparison with respective spikes in the sham condition (F(4,4573) = 4.274, p = 0.002, η^2^ = 0.004; Figure 1E). The decrease was most pronounced after tones presented in the positive peak condition (Holm-corrected post hoc test, p = 0.021, d = −0.138) but was entirely absent for tones presented in the negative peak condition (p = 1.00).

### BECTS is associated with atypical spindle expression

In light of the suppressive effect of auditory stimulation on spike rate and amplitude, in subsequent analyses we aimed to clarify the underlying mechanism. Specifically, we wondered whether the effect is linked to alterations in SO and spindle activity. Recent investigations of BECTS have pointed to differences in spindle properties.^14^^,^^36^ However, the precise nature of these abnormalities shows inconsistencies between the studies. We further asked whether the spike suppression might have been a consequence of generally lightened or disturbed sleep during random stimulation. To this end, as a first step, we compared sleep oscillations between the stimulation conditions and between patient and control groups using EEG power estimates, based on irregular-resampling auto-spectral analysis (IRASA)^37^.

**Figure 2:**
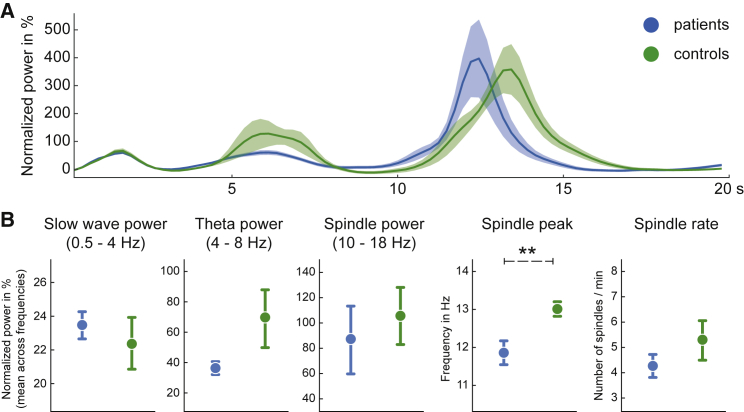
BECTS is associated with slower sleep spindles Results of analysis of IRASA-derived power estimates and spindle detection for patients (blue) and control participants (green). (A) Oscillatory component of IRASA power spectra relative to fractal 1/f component. Note clear peaks in the spectrum for slow wave (0.5–4 Hz), theta (4–8 Hz), and spindle (10–18 Hz) frequency bands. (B) Comparison of slow-wave power, theta power, spindle power, spindle peak frequency, and spindle rate between patients (left) and controls (right). Sleep spindles reached their peak power at a lower frequency in patients than in controls (p = 0.002). See Table S3 for a tabular listing of all findings related to electrophysiological parameters. Data in (A) were taken from Cz during the sham condition and are represented as mean ± boot-strapped SEM. Data in (B) were used for statistics and taken from both Cz and the detection electrode. ∗∗p < 0.01.

An overall examination of spectral power in sham and random delay conditions revealed distinct peaks in the 0.5 to 4 Hz slow wave, 4 to 8 Hz theta, and 10 to 18 Hz spindle bands in both groups (Figure 2A). Testing across groups (patient/control), conditions (random delay/sham), and electrodes (Cz/DET) revealed that compared to controls, patients showed slightly increased slow-wave power in the random delay stimulation condition at Cz (interaction electrode × condition × group, F(1,12) = 5.406; p = 0.038, η^2^ = 0.003). The analysis did not result in further significant differences in slow-wave power (F < 2.594 and p > 0.133 for all main effects and interactions) or theta power (all F < 2.877 and p > 0.116) between stimulation conditions or groups. Power in the spindle band was, as expected, higher at Cz than at the detection electrode (main effect electrode, F(1,12) = 15.592, p = 0.007, η^2^ = 0.113) and slightly higher during the sham than the random stimulation condition (interaction electrode × condition, F(1,12) = 9.444, p = 0.010, η^2^ = 0.001). Intriguingly, inspection of the spectra showed that the spindle peak was at a slower frequency in patients than in the controls (patients: 11.69 ± 0.15 Hz; controls: 13.27 ± 0.22 Hz; main effect group, F(1,12) = 14.532, p = 0.002, η^2^ = 0.389, Figure 2B). Moreover, independent of the group, in the random delay condition, the spindle peak frequency was slightly faster than in the sham condition (random delay: 12.57 Hz; sham: 12.40 Hz; main effect condition, F(1,12) = 7.543, p = 0.018, η^2^ = 0.004), suggesting externally elicited spindles to be faster than spontaneously occurring spindles. Analyzing the occurrence of detected events revealed higher SO rates after random stimulation (F(1,12) = 7.533, p = 0.018, η^2^ = 0.048). The spindle rate was higher at Cz than the detection electrode (main effect electrode, F(1,12) = 9.217, p = 0.010, η^2^ = 0.094; see Table S3 for a tabular listing of these findings and Figure S2 for a spectral analysis of recordings at Fz). In summary, there were distinct electrophysiological differences between stimulation conditions as well as between the patient and healthy control groups, with the most relevant findings concerning sleep spindles. These results led us to focus subsequent analyses on sleep spindles as the main candidate for conveying the suppressing effect on spike rates.

### Competitive relationship between spikes and spindle activity

**Figure 3:**
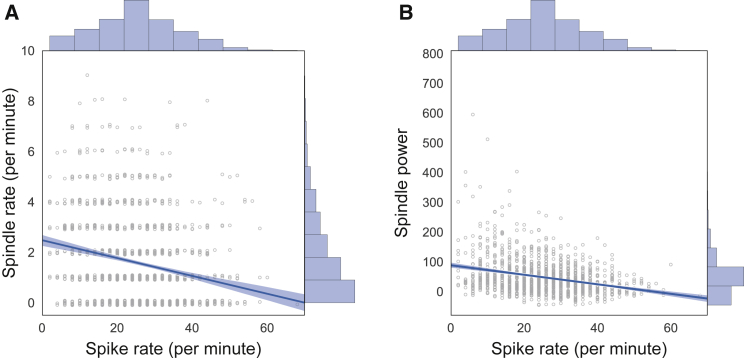
Spike rate is negatively correlated with spindle rate and power Across all conditions, higher spike rates were associated with lower spindle power and rate, pointing to a competitive relationship between epileptic activity and sleep spindles. Each circle represents a single 30 s stimulation block, the regression line is shown ± boot-strapped 95% confidence interval (both Pearson r < −0.231 and p < 2.019 × 10^−17^). Rates are integers leading to largely overlapping data points. To increase clarity, values are slightly jittered along the y axis and marginal histograms illustrate the distribution of values across each axis. Data were taken from the detection electrode.

Considering the alterations in spindle expression in our patients as well as the idea of a potential overlap in spindle-and spike-mediating circuits,^9^^,^^14^, ^15^, ^16^, ^17^ we followed the question of whether tone-induced spindles might affect spikes by occupying thalamocortical networks, thereby suppressing spike promotion. To test this hypothesis, we correlated the rate of spikes with spindle power and spindle rates within 30 s stimulation blocks pooled across patients (Figure 3). This analysis revealed moderate but highly significant negative correlations for all stimulation conditions (r < –.231 and p < 2.019 × 10^−17^ for spindle power and spindle rate correlations across all conditions; all r < −0.135 and p < 0.023, uncorrected, for correlations within individual conditions; values taken from detection electrode), which is consistent with our hypothesis of a principal competitive relationship between spike and spindle activity.

### Spikes and tones evoke comparable spindle activity in patients and controls

**Figure 4:**
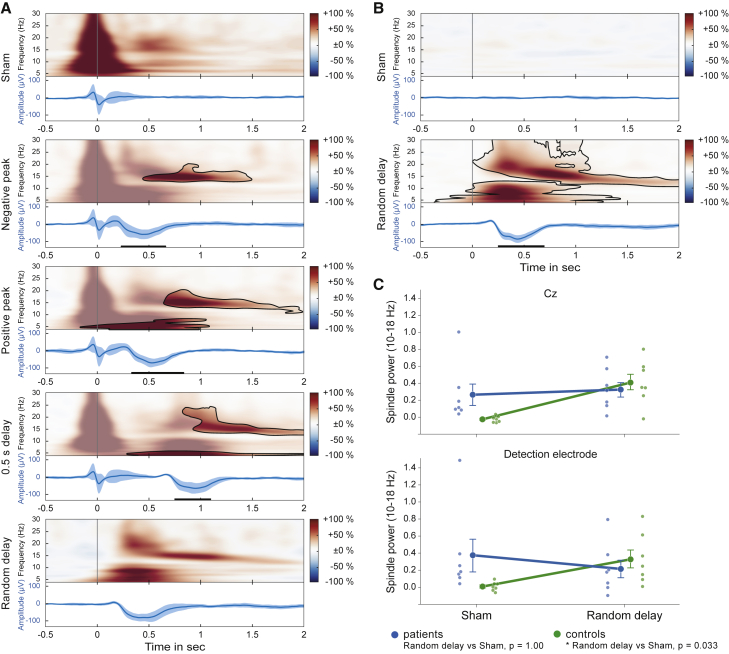
Neural responses to tones in patients and healthy control participants (A) Neuronal responses to spike-triggered auditory stimulation in the patient group, aligned to detected spikes (or tone in random delay condition) at time 0. Each panel shows the time-frequency representation (top; colors denote percent change relative to baseline −1.5 to −0.5 s before the spike negative peak) and potential time-domain representation (bottom; bands show 95% confidence interval) of the evoked response (749.86 ± 108.87 trials per participant; 149.97 ± 0.91 trials per condition). Responses in the negative peak, positive peak, and 0.5 s delay conditions were statistically compared to sham (power: black contours show significant clusters, non-significant areas are covered by a half-transparent mask; time-domain: horizontal bars show significant clusters, all cluster level p < 0.05). Data were taken from Cz. (B) Neural responses in the control group (561.57 ± 24.71 trials per participant; 280.79 ± 7.63 trials per condition). Note that no epileptic spikes were detectable in this group. (C) Spindle responses (averaged 10 to 18 Hz power modulations from 0.4 to 1.2 s after a spike; mean ± boot-strapped SEM) differed significantly between groups and conditions (ANOVA group × sham/random delay, p = 0.019). In the random delay condition, spike-evoked responses approximately cancel out and tone-evoked spindle power was largely equivalent between groups (Holm-corrected post hoc test, patients versus controls, p = 1.00). As expected, the control group showed no spindle response to the sham stimulation, whereas in the patient group, spike-evoked spindle responses in this condition were of the same magnitude as the pure auditory-evoked spindle responses derived from the random condition (random delay versus sham, p = 1.00).

Spikes and spindles being negatively correlated renders spindle activity a candidate for mediating the suppressive effects of stimulation on spike activity. Interestingly, we found that epileptic spikes evoke spindle activity with similar spectral properties as spindles evoked by tones. Figure 4 shows time-frequency-resolved event-related EEG responses to spikes and tones in both patients (Figure 4A) and controls (Figure 4B). Note that in these analyses, power in the sham condition reflects the pure response to the spike in the absence of a tone. In the “peri-spike stimulation conditions” (i.e., the negative peak, positive peak, and 0.5 s delay conditions), the analyzed power results from a combination of spike- and tone-evoked spindle responses. In the random delay condition, spike-evoked responses approximately average out to zero, resulting in the pure neural response to the tone. We isolated evoked spindle power, defined as the evoked power increases between 10 and 18 Hz from 0.4 to 1.2 s after a spike (Figure 4C). The patient group (Figure 4C, blue line) showed a substantial spike-evoked spindle response in the sham condition, which was of the same size as that of the pure auditory-evoked response in the random delay condition (random delay versus sham, Holm-corrected p = 1.00). When comparing patients and healthy controls, spindle power was largely equivalent in the random delay conditions, in which spike-evoked responses in the patient group approximately cancel out and in which spindle power in both groups is mainly the result of the tone (Holm-corrected post hoc test, patients versus controls, p = 1.00). As expected, spindle response to sham stimulation in the control group resembled baseline levels and was significantly lower than in the random delay condition (random delay versus sham, Holm-corrected p = 0.033, d = 0.903; ANOVA group × sham/random delay, F(1,12) = 7.321, p = 0.019, η^2^ = 0.117).

We next examined whether stimulation affected spike-evoked spindle activity, in addition to affecting the occurrence of spikes itself. Such interaction would likely occur in the peri-spike stimulation conditions, in which the tone closely followed the detected spike (i.e., the negative peak, positive peak, and 0.5 s delay conditions). Comparing post-spike spindle responses across conditions revealed significant differences in power (ANOVA sham/negative peak/positive peak/0.5 s delay, main effect condition, F(3,18) = 5.634, p = 0.007, η^2^ = 0.255), with higher spindle-related power in the peri-stimulation conditions (Figure S3, gray lines). However, after subtracting tone-evoked power from these conditions, spindle-related power did not differ anymore from those in the sham condition (main effect condition, F(3,18) = 0.202, p = 0.752; Figure S3, red lines). This outcome of comparable spindle power response after removal of tone-evoked power indicates that in the peri-spike stimulation conditions, spindle responses represent a mere superposition of the responses evoked by spikes and tones in isolation.

## Discussion

We examined the effects of different protocols of auditory stimulation (peri-spike, random delay, and sham) on interictal epileptic discharges (spikes) during NonREM sleep in children with BECTS. We found that tone stimulation generally diminished spike rates, with this effect being most robust in the random delay condition, in which the tone followed the spike with a considerable delay (1.5–3.5 s). Moreover, spikes following a tone within 1.5 s were reduced in amplitude, except for tones presented during the hyperpolarizing (negative) phase of the spike (i.e., conditions of network inactivation). Independent of the stimulation protocol, BECTS patients showed distinct alterations in sleep EEG activity, i.e., distinctly lowered spindle peak frequencies, in comparison with age-matched controls. Moreover, spindle rate and power were negatively correlated with spike rates across all stimulation conditions in the BECTS patients. These findings support the assumption that spindle and spike generation rely on interconnected thalamocortical networks that may also mediate the effects of auditory stimulation.

### Mechanisms

There is evidence that spike formation involves thalamocortical networks^9^^,^^14^, ^15^, ^16^, ^17^ and thalamocortical circuits also participate in the expression of SOs^19^^,^^20^ and sleep spindles^18^ during NonREM sleep. Moreover, auditory stimuli presented during NonREM sleep typically induce a SO that nests a spindle in its up state. Specifically, the tone response begins with a down state^34^ that is associated with thalamic hyperpolarization and followed by rebound bursting at the spindle frequency in thalamocortical cells.^31^^,^^38^ Against this backdrop, we hypothesized that effects of auditory stimulation on spikes during NonREM sleep rely on overlapping thalamocortical networks driving both spike and spindle activity. This idea is indeed corroborated by our present findings. BECTS patients in the present study exhibited abnormalities in the expression of spindles (lowered peak frequency), a phenomenon which has been similarly observed in a recent study on thirty BECTS patients.^14^ In addition, spike rates in our BECTS patients were robustly negatively correlated with spindle power and spindle rate. The negative direction of the correlations indicates that the processes involved in spikes and spindles are competitive in nature. Interestingly, total spike suppression was most pronounced during random delay stimulation (with a delay of 1.5 to 3.5 s between spike and tone), which indicates that close temporal proximity between tone and spike is not a prerequisite for the effect. We propose refractoriness as a potential mechanism that could plausibly explain these temporal dynamics. Refractory periods have been demonstrated for interictal spikes.^39^ Likewise, there is evidence that sleep spindles show refractory periods of up to several seconds,^40^^,^^41^ resulting from a hyperpolarization-activated cation current (Ih), which downregulates the excitability of intrathalamic networks.^41^^,^^42^ In line with previous studies and our own data, we propose that auditory stimulation during sleep is a way to produce spindle refractoriness.^43^ We speculate that presenting tones might mimic the effect of spontaneous spindle activity, in that they excite extensive spindle-generating thalamocortical networks that also encapsulate more localized spiking circuits. According to this idea, tones would induce refractory periods in both these circuits, compatible with the two main effects observed in this study: spike reductions being most robust for random delay stimulation (where the overlap with spontaneously induced refractory periods is minimal), as well as the consistent negative correlation between spike and spindle rates.

The explanation of competitive refractoriness in thalamocortical networks driving both the occurrence of spikes and spindles appears to be also compatible with our finding that tones presented shortly after a detected spike (in the peri-spike stimulation conditions) did not alter spike-evoked spindle activity. While this negative finding was unexpected, it points toward an all-or-nothing response to successfully formed spikes. This means that spikes seem to irreversibly activate the spindle-expressing neuronal machinery before any tone-elicited response can interfere with the respective networks. Importantly, the suppressive effects of stimulation on spike activity were not simply due to a lightening or disturbance of sleep. Random delay stimulation even enhanced slow oscillatory activity, which is a sign that stimulation may have deepened sleep.^44^

### Clinical relevance

Reduction of spike activity by random delay stimulation with tones was found in each of the seven patients, with the decrease in spike rates ranging from 2.4% to 27%. While the magnitude of the suppression at the lower end of this range may seem moderate in comparison with pharmacological treatment, auditory stimulation, as used in the current study, can be assumed to be low in adverse effects. Auditory stimulation may deepen sleep and, if applied over a longer period, may be suitable to attenuate the suspected negative effects of spike activity on cognitive functions. Moreover, it is reasonable to assume that our findings of stimulation-induced spike suppression provide a low estimate on the technique’s potential efficacy. During the stimulation period, only around one third of all spikes triggered a stimulation. The stimulation period itself only covered less than one quarter of the entire night. This leaves substantial room for improving the overall effect of our stimulation. Furthermore, in the most effective random delay condition, tones were more likely than in the peri-spike conditions to occur outside the close temporal proximity of a spike. Assuming that such timing most strongly dampens spike-generating networks, taking additional measures to increase the temporal distance from ongoing spikes might further enhance stimulation efficacy. In contrast, it may also be sufficient to ensure NonREM sleep and present tones in a randomized fashion independently of the occurrence of spikes. Whether optimizing the temporal distance to spikes on the one hand or utilizing such a simplified stimulation paradigm on the other hand modulates the efficacy of the stimulation is subject to future investigation. Auditory stimulation may also be transferable to other types of epilepsy, like epilepsy with CSWS, in which interictal spikes have been found to adversely affect physiological sleep rhythms.^13^^,^^45^ Of relevance here is a recent study that explored effects of auditory stimulation during sleep SOs in children with heterogenous epilepsy subtypes.^46^ While findings were overall negative, the study revealed a slight reduction in spike rates in two out of three children receiving tones during identified SO up states, a protocol bearing similarity with the present random delay stimulation condition.

The small sample size represents a clear limitation of the present study. This issue is mitigated, however, by the homogeneity of the sample, which was selected based on strict diagnostic criteria, as well as the consistency of the demonstrated effect. Indeed, we observed a reduction of spike activity in the auditory stimulation conditions in each of the participating patients. Nevertheless, to substantiate their clinical relevance, our findings need to be confirmed in a larger patient cohort. Also, the effects of more extended periods of stimulation and whether they translate into an amelioration of cognitive functions are yet to be studied. In sum, although in need of further confirmation, the present finding of auditory stimulation-induced reductions of epileptic spikes in children with BECTS seem promising. Our results may open an avenue for the development of stimulation-based approaches to the treatment of childhood epilepsies that might eventually complement or even replace pharmacological treatments.

### Limitations of the study

The present study was performed on a small sample. Large multi-center studies are needed to corroborate our findings. Furthermore, stimulation was performed for only a few hours. To investigate potential effects of spike suppression on the cognitive deficits seen in BECTS patients, stimulation needs to be performed over extended time periods under regular assessment of the affected cognitive abilities. As a last point, while the demonstrated effects were robust across participants, the overall effect size was moderate. This study was not designed to optimize spike suppression but to minimize the burden on the child and maximize our gain in scientific insight into the physiological underpinnings of the effect. As a result, stimulation rate and volume were kept rather low, and the only parameter adjusted between experimental conditions was the stimulation delay after detecting a spike. The employed auditory stimulation technique offers a wide parameter space, which may allow for stronger suppression effects and should be explored in future studies.

## STAR★Methods

### Key resources table

**Table:** 

REAGENT or RESOURCE	SOURCE	IDENTIFIER
**Deposited data**		

All electrophysiology-derived data	https://osf.io/pd5x7/	N/A

**Software and algorithms**		

JASP 0.13	https://jasp-stats.org	N/A
MATLAB 2017a	Mathworks, Natick, USA	N/A
Fieldtrip	https://www.fieldtriptoolbox.org	N/A
Python 3.7	https://www.python.org/	N/A
Seaborn 0.9.0	http://seaborn.pydata.org/	N/A
Spike2 version 7	Cambridge Electronic Design, Cambridge, UK	N/A

### Resource availability

#### Lead contact

Further information and requests for resources should be directed to the lead contact, Jens G. Klinzing (jens.klinzing@uni-tuebingen.de).

#### Materials availability

This study did not generate new unique reagents.

### Experimental model and subject details

A total of 14 human subjects (7 girls, 7 boys; mean age ± SD: 9.97 ± 1.52; range: 6.60 - 11.76 years) participated in the study. Half of the participants (“patients”) had previously been diagnosed with BECTS or BECTS-typical centrotemporal spikes and did not have any known structural neuronal abnormalities. The other participants (“controls”) had not been diagnosed with epilepsy or any other known neurological disorders. Controls were matched to the patients by age (patients: 9.86 ± 1.65 years; controls: 10.08 ± 1.50 years; p = 0.798) because the investigated form of epilepsy as well as physiological sleep characteristics change substantially with age. Four of the patients were on epilepsy medication. See Table S1 for details. Sample size was a result of patient availability. Two additional participants were excluded from analysis due to technical problems during the experimental night. Further four subjects were excluded who had previously presented with BECTS-typical centrotemporal spikes but showed no or almost no epileptic activity during the experimental night. Potential patients were preselected, approached, and informed about the study by an experienced pediatrician (S.R.) with access to the candidate’s medical history. Potential control participants were contacted via the institute’s volunteer database. Exclusion criteria were other neurological or psychological disorders, irregular sleeping patterns, or ongoing participation in other studies. In accord with the Declaration of Helsinki, the participants’ parents gave their written informed consent, subjects gave their verbal consent, and both were free to abort the study at any stage. The study was approved by the local ethics committee of the Medical Faculty of the University Tübingen.

### Method details

#### Experimental procedure

Accompanied by one parent, participants arrived at the laboratory (Institute of Medical Psychology and Behavioral Neurobiology, Tübingen, Germany) at around 7:00 pm. EEG and other polysomnographic electrodes were attached, earphones were put on, the individual hearing threshold was determined (43.00 ± 2.13 dB sound pressure level, mean ± SEM), and sleepiness was rated verbally on the Stanford Sleepiness Scale (no difference between groups, p = 1.00). At around 10:00 pm, participants went to bed. Parents either slept in the same room as the child or in a room next door. After the stimulation period (see below), participants continued sleeping without further interventions. Subjects were kept blind with respect to the exact nature of the stimulation and different conditions. Recruitment and data collection were performed between April 2017 and September 2019.

#### Electrophysiological and sleep recordings

Throughout the night, we acquired electroencephalographic recordings at 19 electrode sites (Fp1, Fp2, F3, Fz, F4, F7, F8, C3, Cz, C4, T3, T4, T5, T6, P3, Pz, P4, O1, and O2, according to the International 10-20 system), referenced to the mastoids (averaged M1, M2). Additionally, we obtained bipolar electromyographic (EMG) recordings from the chin as well as horizontal and vertical electrooculography (EOG) to aid sleep scoring. Data were recorded and amplified with a Brain Products recording system (Brain Products GmbH, Gilching, Germany) at a sampling frequency of 500 Hz and processed using MATLAB 2017a (Mathworks, Natick, USA), Fieldtrip (fieldtriptoolbox.org), ^47^ Python 3.7, Seaborn 0.9.0 (seaborn.pydata.org). In most cases, analysis was restricted to the Cz electrode site as well as the individual detection electrode (DET) nearest to the focus of the patient’s spike activity. For control subjects, the same electrode as the age-matched patient was chosen as detection electrode. Sleep recordings were scored based on C3, C4, EMG, and EOG, according to standard criteria^48^ as sleep stages S1–S4, REM sleep, and wakefulness. Sleep architecture did not differ between patients and controls (all p > 0.15, Table S4).

#### Auditory stimulation

The EEG electrode closest to the previously determined epileptic focus was used to detect epileptic spikes (see Table S1). The signal from this detection electrode (DET) was passed on to the stimulation setup, referenced to the EEG system’s mastoid electrodes. In one patient where spikes were found to be distinctly more pronounced at another electrode in the very beginning of the experimental recordings, the detection electrode was switched to this other site before the patient was firmly asleep and before stimulation was started. Activity at the detection electrode was recorded by a Digitimer D360 EEG amplifier (Digitimer Ltd., Welwyn Garden City, UK). Using in-built hardware filters (corresponding to a Butterworth filter of order 2), this signal was bandpass filtered between 4 and 150 Hz. This range was chosen to remove the dominant slow wave activity while extracting the broad spectral range of epileptic spikes. The analog DET signal was sampled at a rate of 200 Hz by a CED Power1401 MK2 data acquisition interface (Cambridge Electronic Design, Cambridge, UK). Importantly, a built-in sequencer unit allowed a real-time processing of the incoming filtered and digitalized DET signal. Spike detection was implemented via a custom-made script running under Spike2 version 7 (Cambridge Electronic Design, Cambridge, UK) using a threshold procedure.^34^ The script triggered auditory stimulation consisting of a burst of pink noise (50 ms duration, 5 ms falling and rising flank, +12 dB above previously established hearing threshold), which were administered using in-ear headphones. Detection threshold and frequency range for the online filtering were continuously adapted to maximize spike detections (true positives) while minimizing false detections of other events (false positives).

We performed auditory stimulation during NonREM sleep (stages S2-S4) in five conditions, which were randomly switched in blocks of 30 s (Figure 1A). These conditions were designed to allow investigating the influence of stimulation on both, already ongoing as well as subsequently emerging epileptic activity. Tones were presented either at the time of the negative peak of a detected spike (“Negative peak”), or 90 ms later during its subsequent positive peak (“Positive peak”), or 0.5 s after the negative peak (“0.5 s delay” condition), or with a random delay between 1.5 and 3.5 s after the negative peak (“Random delay”). The Random delay condition thereby replaced a fully random condition with the benefit that stimulation was performed in the temporal context of detected spikes. This excluded a scenario in which many stimulations are performed during periods that differ vastly in epileptic activity from the other conditions. In a “Sham” control condition, the negative peak of a spike was detected but no auditory stimulation was performed. After each stimulation, detection of further spikes was paused for 2.5 s to allow for analysis of the evoked response. In the control subjects, in which there were no spikes to time the stimulation to, the Random delay and Sham conditions were based on random time points during NonREM sleep instead of negative peaks. Data from the control group were acquired to detect potential baseline differences or influences of ongoing epileptic activity on auditory processing during sleep.

Stimulation was paused at signs for arousal, awakening, or REM sleep. Auditory stimulation was started as soon as the polysomnography indicated reliable sleep patterns and continued for about 3 h (174.14 ± 13.94 min, mean ± SEM). Depending on the participant’s sleep pattern and frequency of detectable spikes, this resulted in 183.19 ± 18.48 stimulations per subject and condition. The ratio of stimulation blocks applied during S2 versus SWS was not significantly different between stimulation conditions (ANOVA main effect Condition, p = 0.423), which is an expected result of the randomized selection of stimulation conditions during the experiment. Of note, only ∼2% of blocks overlapped with Wake, N1 or REM sleep.

#### Data analysis

##### Offline spike detection and removal by EEG signal interpolation

We detected interictal epileptiform discharges automatically using custom-made algorithms. We first performed an Independent Component Analysis (ICA) decomposition (fastICA) and manually selected ICA components that did not contain spike-like waveforms for rejection. After a back-projection into EEG space, we highpass-filtered the EEG signal of the detection electrode at 5 Hz, extracted the envelope using a Hilbert transform, and smoothed the envelope with a moving average of 50 ms length. For each subject, we determined the detection threshold as the mean +2.5 times the standard deviation (SD) of the envelope signal over all NonREM periods. For one subject, the scaling factor was adjusted to 2.0. A spike was registered whenever the enveloped exceeded the threshold for more than 10 and less than 500 ms. Putative events following within < 50 ms were merged. For each spike event, we marked on- and offset where the envelope crossed the threshold. All spikes were visually validated before further analysis. We then calculated the spike rate, i.e., events per minute, separately for each experimental condition. To assess differences in spike rate between condition, we pooled rates for all 30 s stimulation blocks across patients and then performed an Analysis of Variance (ANOVA) with a fixed Factor ‘Condition’ (Negative peak/Positive peak/0.5 s delay/Random delay/Sham) and spike rate as the dependent variable. See Table S2 for spike rates for each patient and condition.

We assessed the effect of auditory stimulation on spike amplitude by selecting for each condition all spikes occurring within 1.5 s after stimulation. Amplitude was determined by calculating the distance between a spike’s most negative trough and its most positive peak. To account for inherent inter-individual amplitude differences, spike amplitudes were z-scored across all conditions for each patient. Statistical differences were examined by pooling spike amplitudes across patients and performed an ANOVA with a fixed factor ‘Condition’ (Negative peak/Positive peak/0.5 s delay/Random delay/Sham) and spike amplitude as the dependent variable.

Finally, to remove confounding effects of spikes in subsequent analyses, we created a spike-free dataset by performing a spline interpolation on the EEG time-domain signal for each identified spike with additional padding of 100 ms on each side (Figure S1; see Figure S2 for a power analysis before/after spike removal).

##### Spectral analysis

To assess spectral power (for patients on the interpolated signal), we segmented data for each experimental condition into epochs of 4.096 s with an overlap of 50%. We estimated the frequency content of the signal using the irregular-resampling auto-spectral analysis (IRASA) approach.^37^ This procedure allows the isolation of oscillatory spectral components from fractal 1/f background component typical for electrophysiological recordings. We expressed the magnitude of the oscillatory component relative to the fractal component. Power was assessed by averaging the resulting values for the slow wave (0.5-4 Hz), theta (4-8 Hz) and spindle (10-18 Hz) bands, respectively. Peak spindle frequencies were determined individually for each subject. We assessed statistical differences in theta and spindle power as well as spindle peak frequencies by performing a repeated-measures ANOVA with between-subject factor ‘Group’ (Patients/Controls) and within-subject factors ‘Electrode’ (Cz/DET) and ‘Condition’ (Random delay/Sham).

##### Offline detection of SOs and sleep spindles

Discrete slow oscillation and spindle events were detected during NonREM epochs across the entire night.^49^ For SOs, each EEG channel was first bandpass-filtered from 0.3 to 1.25 Hz. Then positive to negative zero crossings were identified and all intervals between consecutive zero crossings shorter than 0.8 or longer than 2 s (corresponding to frequencies of 0.5-1.25 Hz in the SO range) were discarded. Across the remaining intervals, the negative peaks and the amplitude from negative to the positive peak were averaged. In line with previous work,^35^^,^^50^ the resulting mean values were multiplied by 1.5 and served as detection threshold, i.e., intervals were labeled as a SO whenever (i) its negative peak was lower than 1.5 times the mean negative peak value and (ii) the amplitude exceeded 1.5 times the mean amplitude threshold. To detect sleep spindles, we bandpass-filtered the EEG data between 10 and 18 Hz and derived the root-mean-square (RMS) of the signal with a 200-ms sliding window, followed by smoothing with an identical window length. We determined a detection threshold from the mean RMS signal across all NonREM epochs +1.5 times its SD. Based on this threshold, intervals for which the RMS signal exceeded the threshold for more than 0.4 s and less than 3 s were labeled as discrete spindle events. Time points exceeding an upper threshold determined by the mean RMS signal plus 5 times its SD were excluded.^51^ We determined SO and spindle rate, i.e., the number of detected events per minute, separately for each experimental condition. Statistical assessment of SO and spindle rate was based on a repeated-measures ANOVA with the between-subject factor ‘Group’ (Patients/Controls) and the within-subject factors ‘Electrode’ (Cz/DET) and ‘Condition’ (Random delay/Sham). We further examined how sleep spindles relate to spikes by estimating the rate, i.e., events per minutes, of detected spindles and spindle power (10-16 Hz) for all 30 s stimulation blocks. We correlated spindle rates and spike rates (for the same blocks) across all patients.

##### Analysis of evoked responses

For analyses of spike- and stimulation-related activity, non-interpolated data were cut into segments of 14 s around detected spikes. Segments containing artifacts were rejected using a semi-automatic procedure (identification of candidate artifacts by thresholding the z-transformed signal, followed by thorough visual inspection). Data were highpass-filtered at 0.1 Hz and notch-filtered between 45 and 55 Hz (Butterworth filter). Evoked potentials were corrected by subtracting an average baseline value between −1.5 and −0.5 s before the detected spike. For time-frequency analyses, 12-cycle Morlet wavelets were applied and visualized as relative change, i.e., power change relative to the average power at that frequency between −1.5 and −0.5 s. Statistical analyses for evoked potentials and evoked time-frequency response relied on non-parametric cluster-permutation statistics to control inflation of type I errors due to multiple comparisons.^52^ For time-frequency data, samples were selected that showed significant differences in power in relation to the respective contrast condition (two-tailed paired-samples t tests, sample-level alpha = 0.05). In the resulting statistical map, adjacent samples were grouped into positive and negative clusters for which cluster-level statistics were calculated by summing up the t-values within each cluster. These were tested against a reference distribution (cluster-level alpha = 0.05), generated by shuffling the association of data and condition (10,000 permutations) and, for each permutation, taking the maximum statistics among all clusters.

In a separate step, we isolated evoked spindle power by averaging time-frequency data over all samples within a spectral-temporal segment after applying a broad Tukey window (α = 0.25) to attenuate power at the edges. The analyzed segment corresponded to power occurring in the 10-18 Hz spindle band and 0.4-1.2 s following spike onset (negative peak). The spectral and temporal width of the window was determined based on the grand average over all control subjects for the Cz electrode. We statistically assessed evoked spindle power by performing a repeated-measures ANOVA with factors ‘Electrode’ (Cz/DET) and ‘Condition’.

### Quantification and statistical analysis

Statistics were calculated using JASP 0.13 (https://jasp-stats.org) and relied on general linear models (GLM), including analyses of variances (ANOVA), t tests and linear correlations. For all statistics, we performed two-tailed tests. A p value < 0.05 was considered significant. Data were inspected for violations of relevant test assumptions. Degrees of freedoms were Greenhouse-Geisser-corrected in case the assumption of sphericity was violated. Post hoc tests were Holm-corrected for multiple comparisons. Effect sizes (η^2^ for ANOVA, Cohen’s d for post hoc tests, Pearson r for correlations) are provided for significant tests.

## Data Availability

All electrophysiology-derived data required to reproduce our findings (including spike rates, power estimates etc.) as well as all statistical analyses (in the form of JASP analysis files) have been uploaded to the Open Science Framework and are publicly accessible: https://osf.io/pd5x7/ (https://doi.org/10.17605/OSF.IO/PD5X7).

## Supplemental information

Document S1. Figures S1–S3 and Tables S1–S4

Document S2. Article plus supplemental information
