# Supplementary material for: Auditory stimulation during sleep suppresses spike activity in benign epilepsy with centrotemporal spikes
Source: Cell Rep Med. 2021 Oct 26;2(11):100432. doi: 10.1016/j.xcrm.2021.100432 (PMC8606903; doi:10.1016/j.xcrm.2021.100432)
Supplement: Document S1. Figures S1–S3 and Tables S1–S4 [file mmc1.pdf]

Cell Reports Medicine, Volume *2*

Supplemental information

Auditory stimulation during sleep suppresses spike activity in benign epilepsy with centrotemporal spikes

Jens G. Klinzing, Lilian Tashiro, Susanne Ruf, Markus Wolff, Jan Born, and Hong-Viet V. Ngo

Supplemental Information

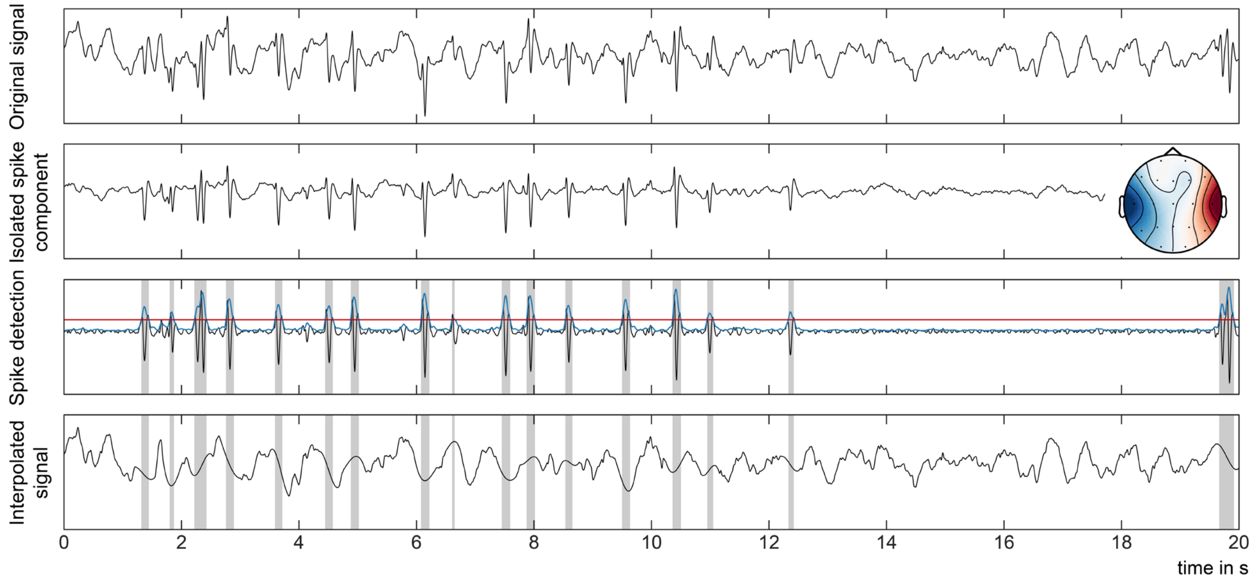

Supplementary Figure 1 Spike detection and EEG signal interpolation. Related to STAR Methods. From the original time-series data (first trace from top), we extracted components showing prominent spikes (second trace, head plot shows highly localized spike topography). After highpass-filtering, the amplitude envelope of the signal was calculated, and an individual threshold (red) was determined to detect spikes (third trace). Detected spikes were removed and the EEG signal was interpolated, resulting in a spike-free signal (fourth trace). Vertical grey bars indicate the on- and offset of detected events.

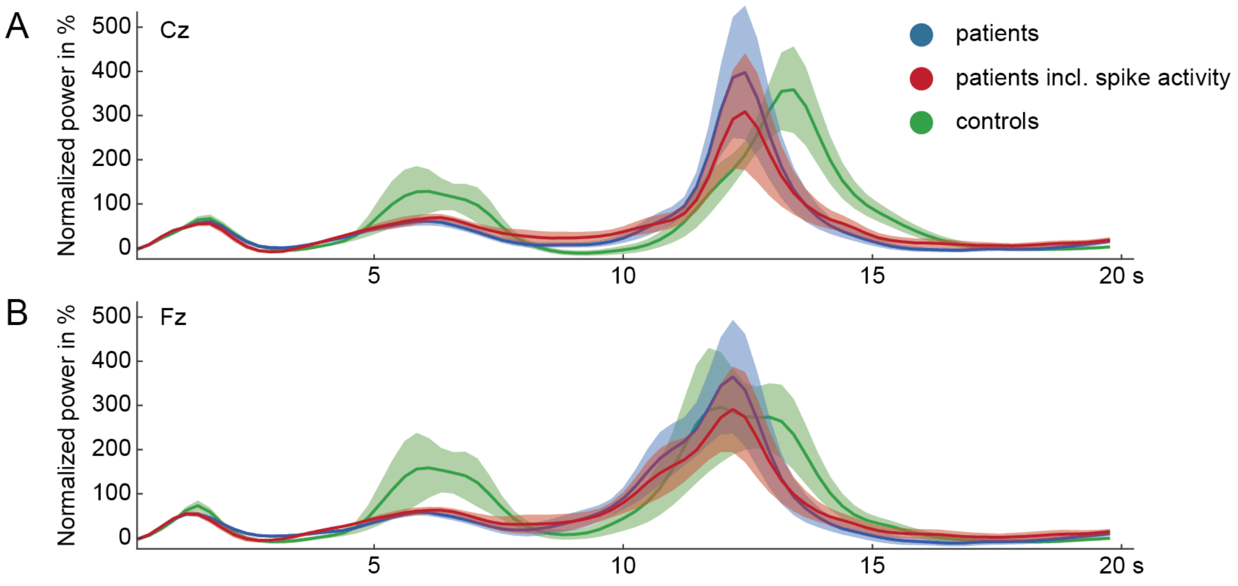

Supplementary Figure 2 Power analysis of frontal activity and non-interpolated signal including spikes. Related to Figure 2 and STAR Methods. IRASA-derived power estimates of the Sham condition as in Figure 2, with additional data shown from electrode Fz (B) and the signal before spike interpolation (red). Note that spike interpolation did not change the frequency of the spindle peaks. In both groups, a second spindle peak at lower frequencies is visible in Fz (B), as expected for frontal slow spindles. In parallel to fast spindles, this slow spindle peak is shifted to slower frequencies in the patient group compared to controls.

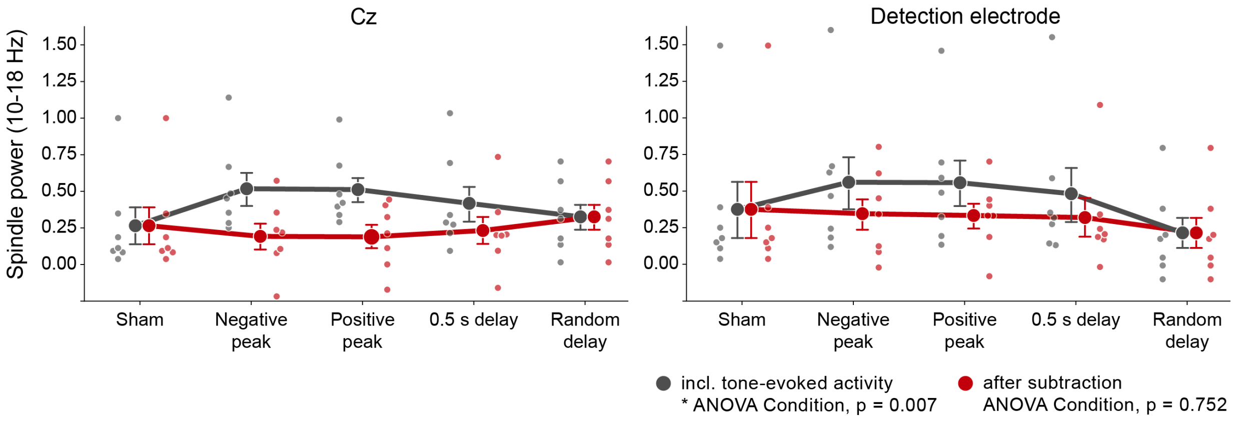

Supplementary Figure 3 Spike- and tone-evoked spindle responses are independent. Related to Figure 4. Evoked spindle power (response averaged between 10 and 18 Hz and from 0.4 to 1.2 s after a spike) differed significantly across the stimulation conditions (grey line, ANOVA Sham/Negative peak/Positive peak/0.5 s delay, main effect Condition, $p = 0.007$). To examine whether higher spindle power in the Peri-spike stimulation conditions (i.e., the Negative peak, Positive peak, and 0.5 s delay conditions) was merely the result of an additive combination of spike- and tone-evoked responses, we subtracted tone-evoked responses (obtained in the Random delay condition, see main text) from the Peri-spike stimulation conditions, before extracting spindle power. This procedure resulted in comparable spindle frequency band power in all conditions (red line, $p = 0.752$). We performed the identical analysis on evoked delta power (2-4 Hz), with very similar results (main effect Condition before subtraction of tone-evoked activity as derived from the Random delay condition, $p < 0.001$; after subtraction, $p = 0.172$). Data are represented as mean ± boot-strapped SEM. See Figure 4 for time-frequency resolved event-related responses from which these values were extracted.

Supplementary Table 1 Participant details.

	Subject	Sex	Age	Detection electrode	Medication
**Patients**	1	m	9.3	T4	Oxcarbazepine
	2	w	10.8	T4	Levetiracetam
	3	w	10.5	T3	None
	4	m	6.6	Cz	None
	5	m	11.8	T4	Sulthiame
	6	w	9.6	C3	Sulthiame
	7	w	10.5	C3	None
**Controls**	8	m	6.9	C3	None
	9	m	9.6	C3	None
	10	m	10.9	Cz	None
	11	w	10.8	C3	None
	12	m	11.4	F4	None
	13	w	10.2	Cz	None
	14	w	10.8	Cz	None

Related to STAR Methods. Participants in both groups were matched by age (difference between groups, $p = 0.798$). The detection electrode was chosen based on previously determined epileptic foci and spike amplitude in the recording night.

Supplementary Table 2 Spike rates for each patient and condition.

**Subject**	**Spike rates per minute in each condition**				
	**Sham**	**Negative peak**	**Positive peak**	**0.5 s delay**	**Random delay**
1	20.6	20.7	20.7	19.8	20.1
2	27.1	27.5	22.6	22.9	19.9
3	31.6	27.6	26.4	30.6	30.5
4	32.7	33.3	32.5	33.1	30.3
5	25.7	26.6	26.9	25.5	24.3
6	24.3	27.3	26.7	24.1	21.6
7	21.0	16.7	19.1	19.9	17.8
Mean ± SEM	26.1 ± 1.65	25.7 ± 1.88	25.0 ± 1.59	25.1 ± 1.78	23.5 ± 1.78

Related to Figure 1 and STAR Methods. The number of spikes was determined for each stimulation block and averaged over all blocks of each condition. Spike rates are provided as number of spikes per minute.

Supplementary Table 3. Electrophysiological parameters.

Parameter	Effect	p	η^2	Description
Spindle power	Electrode	0.007	0.113	Spindle power was higher at Cz than at the detection electrode.
Spindle power	Electrode × Condition	0.010	0.001	Spindle power at Cz was higher during the Sham than the Random stimulation condition.
Spindle rate	Electrode	0.010	0.094	Spindle rate was higher at Cz than the detection electrode.
Spindle peak frequency	Condition	0.018	0.004	Spindles in the Random Delay stimulation condition were faster than in the Sham condition.
Spindle peak frequency	Group	0.002	0.389	Spindles in the Patient group were slower than in the Control group.
Slow wave power	Electrode × Condition × Group	0.038	0.003	Slow wave power in Patients was higher in the Random delay stimulation condition at Cz.
Slow oscillation rate	Condition	0.018	0.048	Higher slow oscillation rate in the Random delay condition than in the Sham condition.

Related to Figure 2. Summary of statistical results from an ANOVA on electrophysiological parameters. The ANOVA included the factors Electrode (Cz/Detection electrode), Condition (Random Delay/Sham) and Group (Patients/Controls). Listed are all significant effects. All results including descriptive plots can be found in the data repository associated with this publication.

Supplementary Table 4. Sleep parameters.

	Subject	S1	S2	S3	S4	REM	TST
**Patients**	1	15.0	204.0	178.5	60.5	40.5	505.0
	2	68.0	390.0	66.0	20.0	95.0	643.0
	3	37.0	214.0	126.0	76.0	87.5	572.0
	4	1.5	47.5	461.0	98.5	16.0	639.0
	5*	24.0	103.5	28.5	57.0	27.0	250.5
	6	20.0	162.5	254.0	53.5	0.0	496.0
	7	23.5	259.0	112.5	57.0	95.0	553.5
Mean ± SEM		27.50 ± 9.37	212.83 ± 46.00	199.67 ± 58.48	60.92 ± 10.62	55.67 ± 17.33	568.08 ± 25.85
**Controls**	8	15.0	151.0	128.0	40	69.0	404.0
	9	59.0	220.0	77.0	65.5	89.5	526.0
	10	51.0	267.5	94.0	70.5	93.0	631.5
	11	8.5	155.0	184.0	101.5	96.5	569.5
	12	65.0	151.0	144.5	55.0	50.5	583.0
	13	31.5	135.0	89.5	69.0	25.5	419.0
	14	26.5	280.5	69.5	63.5	78.5	553.5
Mean ± SEM		36.64 ± 8.31	194.29 ± 23.02	112.36 ± 15.70	66.43 ± 7.06	71.79 ± 9.79	526.64 ± 32.13

Related to STAR Methods. Time spent in each sleep stage (in min). There were no differences between groups in any sleep stage (independent t-tests, all $p > 0.15$ uncorrected, subject 5 excluded from means and statistics). * For participant 5, polysomnographic recordings were only available for the first half of the night. Because the patient had difficulties falling asleep after a bathroom break the EEG was removed; the patient's mother reported normal sleep in the second half of the night. Sleep parameters from this participant were excluded from statistics.
